# The role of cardiovascular magnetic resonance in candidates for Fontan operation: Proposal of a new Algorithm

**DOI:** 10.1186/1532-429X-13-69

**Published:** 2011-11-11

**Authors:** Lamia Ait-Ali, Daniele De Marchi, Massimo Lombardi, Luigi Scebba, Eugenio Picano, Bruno Murzi, Pierluigi Festa

**Affiliations:** 1 Institute of Clinical Physiology - National Research Council (CNR), Ospedale del cuore "G.Pasquinucci" Via Aurelia Sud 54100 Massa, Italy; 2 MRI Lab Fondazione G.Monasterio CNR-Regione Toscana Pisa Via G. Moruzzi 1, 56124 Pisa, Italy; 3 Anesthesia departement, Ospedale del cuore "G.Pasquinucci" Fondazione G.Monasterio CNR-Regione Toscana Via Aurelia Sud 54100 Massa, Italy; 4 Institute of Clinical Physiology - National Research Council (CNR), Via G. Moruzzi 1, 56124 Pisa, Italy; 5 Cardiac-surgery department. Ospedale del cuore "G.Pasquinucci" Fondazione G.Monasterio CNR-Regione Toscana Via Aurelia Sud 54100 Massa, Italy; 6 Pediatric Cardiology department, Ospedale del cuore "G.Pasquinucci" Fondazione G.Monasterio CNR-Regione Toscana Via Aurelia Sud 54100 Massa, Italy

**Keywords:** Fontan, Cardiac CMR, Univentricular heart, Cardiac catheterization

## Abstract

**Background:**

To propose a new diagnostic algorithm for candidates for Fontan and identify those who can skip cardiac catheterization (CC).

**Methods:**

Forty-four candidates for Fontan (median age 4.8 years, range: 2-29 years) were prospectively evaluated by trans-thoracic echocardiography (TTE), Cardiovascular magnetic resonance (CMR) and CC. Before CC, according to clinical, echo and CMR findings, patients were divided in two groups: Group I comprised 18 patients deemed suitable for Fontan without requiring CC; group II comprised 26 patients indicated for CC either in order to detect more details, or for interventional procedures.

**Results:**

In Group I ("CC not required") no unexpected new information affecting surgical planning was provided by CC. Conversely, in Group II new information was provided by CC in three patients (0 vs 11.5%, p = 0.35) and in six an interventional procedure was performed. During CC, minor complications occurred in one patient from Group I and in three from Group II (6 vs 14%, p = 0.7). Radiation Dose-Area product was similar in the two groups (Median 20 Gycm^2^, range: 5-40 vs 26.5 Gycm^2^, range: 9-270 p = 0.37). All 18 Group I patients and 19 Group II patients underwent a total cavo-pulmonary anastomosis; in the remaining seven group II patients, four were excluded from Fontan; two are awaiting Fontan; one refused the intervention.

**Conclusion:**

In this paper we propose a new diagnostic algorithm in a pre-Fontan setting. An accurate non-invasive evaluation comprising TTE and CMR could select patients who can skip CC.

## Background

In patients with a functional single ventricle, a staged surgical approach leading to a Fontan procedure has substantially improved life expectancy and functional status [1,2]. Before Fontan, cardiac catheterization (CC) is routinely indicated to detect the suitability for and risks of Fontan [3] or to identify patients who require additional interventions (either by catheter before surgery or in the operating room concomitant with the Fontan procedure) [4]. However, CC is an invasive tool associated with morbidity and mortality [5-7] and uses ionizing radiation, increasing the patient's long-term risk of cancer [8,9]. Transthoracic Echocardiography (TTE) is widely used in such patients, but in the majority of patients it is not sufficiently exhaustive for a complete assessment of intracardiac and mediastinal vessel anatomy [3,10]. As highlighted by Banka et al. [11], a more comprehensive imaging strategy not based solely on echocardiography should be considered. Cardiovascular Magnetic Resonance (CMR) has gained widespread acceptance in the evaluation of pre- and post-operative congenital heart disease [12], including patients with univentricular circulation [13,14]. A randomized study showed that CMR is a safe and cost-effective alternative to catheterization in selected patient candidates for univentricular surgical palliation, before performing bidirectional Glenn as a first step [15]. Previous retrospective studies also suggest that CC could be avoided before Fontan in a certain percentage of patients without adversely affecting outcome [4,16]. Prakash et al. have recently proposed a new diagnostic algorithm to avoid CC in ''low-risk'' subjects before a Fontan operation [16]. Our hypothesis is that a "one-fits-all" approach can be misleading in Fontan candidates, who may benefit most from a tailored approach, restricting CC only to a subset of patients in whom a combined clinical, TTE and CMR approach is inadequate. To test this hypothesis, we initiated a prospective, observational, single-center study, systematically evaluating all Fontan candidates by means of clinical, non-invasive (TTE, CMR) and CC evaluation, in order to propose a tailored diagnostic algorithm and to define patients who could skip CC.

## Method

From June 2002 to March 2011, all candidates for the Fontan operation presenting at the Massa Heart Hospital were prospectively enrolled in this study approved by the ethical committee of our institute. Informed consent was obtained from all parents or legal guardians. All patients (n = 44, median age 4.8 years, range 2-29) resulted eligible for the study. Demographic and clinical features are detailed in Table 1. Major extracardiac disorders were hydrocephalus with myelomenigocele in one patient and right lung hypoplasia in the context of a scimitar syndrome in a second patient.

**Table 1:** Demographic, history and clinical data

	All (44 pts)	Group 1 (18 pts)	Group 2 (26 pts)	P
**Age at enrollment (years)**	4.8 (2-29)	4.7 (2.7-13.6)	5 (2-29)	0.7

**Weight at enrollment (Kg)** **BSA at enrollment (m^2^)**	17 (12-76) 0.72 (0.5-1.8)	19 (14-36) 0.72 (0.6- 1.17)	17 (12-76) 0.71 (0.55-1.8)	0.6 0.3
**Diagnosis:**				
HLHS	6	0	6	
TA	9	6	3	
Ebstein	2	2	0	
DILV	5	2	3	
Single right ventricle	3	2	1	
Unbalanced AV canal	6	.	6	
Complex 2 ventricles	13	6	7	
**Previous CC**	41	18	23	
**Previous surgery**	42	17	25	
**Aorto-pulmonary shunt**	20	10	10	
**Pulmonary artery banding**	10	2	8	
**Cavo-Pulm. Anastomosis**	41	17	24	
**Atrioseptectomy**	14	5	9	
**Norwood**	6	0	6	
**Other^§^**	8	1	7	
**Antegrade pulmonary flow**	17	8	9	
**Peripheral O2 sat (%)**	81 ± 5.8	82 ± 5.8	81 ± 5.9	0.6

Age, weight and BSA are expressed as median and range. ^§ ^Other procedures: 1 tricuspid valve surgical closure, 2 aortic coarctatectomies, 2 total anomalous venous return corrections, 1 ventricular septal defect enlargement, 2 surgical occlusions of significant veno-venous collaterals. AV: atrioventricular; BSA: body surface area; CC: cardiac catheterization; DILV: double inlet left ventricle; HLHS: hypoplastic left heart syndrome; Sat: saturation. TA: Tricuspid atresia

### Study protocol

Histories including surgical and catheterization procedures were retrospectively recorded. All patients were then prospectively evaluated clinically and by laboratory setup including TTE, CMR and CC.

*Echocardiography*: a standard comprehensive TTE exam was performed with an appropriate probe (3 or 5 MHz) according to body weight. Cardiovascular anatomy and function were evaluated including assessment of interatrial and ventricular septum, atrio-ventricular valve morphology and function, pulmonary and systemic venous return, superior cavo-pulmonary anastomosis and pulmonary artery anatomy and flow pattern, ventricular anatomy and function (based on visual quantification), and systemic pathway (outflow tract, aortic valve and aortic arch) anatomy and flow (See Table 2). If an echo did not cover all the abovementioned features it was considered not completed.

**Table 2:** ECHO, CMR and CC data and comparison between the two groups

		All (44 pts)	Group 1 (18 pts)	Group 2 (26 pts)	P
Echo	*AV valve regurgitation:*				
	- Absent/trivial	20	10	10	
	- Mild/Moderate	18	8	10	
	- Severe	6	0	6	
	*ASD:*				
	- Large	37	13	24	
	- Restrictive/absent	4	4	0	
	- Not visualized	3	1	2	
	*Ventricular function:*				
	- Normal	41	18	23	
	- Mild/moderate dysfunction	2	0	2	
	- Severe dysfunction	1	0	1	
	*Systemic obstruction*	5	0	5	
	*Right pulmonary artery*				
	- Not visualized	18	5	13	
	- Stenosis/hypoplasia	3	1	2	
	*Left pulmonary artery*				
	- Not visualized	29	7	22	
	- Stenosis/hypoplasia	2	1	1	
	*Superior vena cava*				
	- Not visualized	7	1	6	
CMR	End ventricular vol. (ml/m^2^)	106 ± 28	97 ± 23	112 ± 30	0.1
	Ventricular EF (%)	61 ± 9.3	64.5 ± 8.5	58 ± 8.9	0.003
	RPA diameter (mm)	12 (6-25)	13 (8-24)	11 (8-21)	0.25
	LPA diameter (mm)	10.5 (5-23)	11.5 (8-22)	9 (5-23)	0.1
	McGoon index	1.98 (1.1-3.4)	2.1 (1.58-2.8)	1.9 (1.1-3.4)	0.8
	Nakata index (mm^2^/m^2^)	237 (107-654)	236 (146-567)	241(107-654)	0.85
	RPA flow (ml/min)	886 (195-3815)	788 (195-3815)	972 (325-2352)	0.9
	RPVs flow (ml/min)*	1573 (900-3900)	1712 (1033-3900)	1403 (900-3100)	0.3
	LPA flow (ml/min)	732 (100-1858)	765 (263-1858)	467 (100-1463)	0.1
	LPV vein flow (ml/min)*	1185 (583-2500)	1250 (745-2360)	1120 (583-2500)	0.6

CC	Radiation DAP (Gycm^2^)	21.6 (5-270)	20 (5-40)	26.5 (9-270)	0.35
	Minor Complications	4	1	3	0.8
	RPA pressure (mmHg)	12.2 ± 2.98	11.2 ± 2.4	13.1 ± 3.4	0.04
	LPA (mmHg)	11.7 ± 3	10.8 ± 2	12.4 ± 3.3	0.05
	SVC pressure (mmHg)	12.8 ± 3	11.3 ± 2.5	13.5 ± 2.5	0.01
	Atrial pressure (mmHg)	8.1 ± 2.2	7.7 ± 2.3	8.5 ± 2.2	0.2
	End ventricular pressure (mmHg)	8.8 ± 2.3	8.5 ± 2.3	9.1 ± 2.3	0.3
	PVR (UI/m^2^)	1.75 ± 0.6	1.55 ± 0.6	1.84 ± 0.46	0.08
	McGoon index	1.95 ± 0.47	1.97 ± 0.4	1.92 ± 0.5	0.7
	Nakata index (mm^2^/m^2^)	275 (77-1044)	325 (158-1044)	257(77-586)	0.3
	Mayo clinic index	2.93 ± 0.94	2.75 ± 1,15	3 ± 0.8	0.9

Continuous data are expressed as mean ± SD and as median and range depending upon the normality of the data. * Assessed in the last 21 patients

Legend: ASD: atrial septal defect; DAP: Dose Area Product; Ed: End-diastolic; EF: ejection fraction ; LPA: left pulmonary artery; LPVs: left pulmonary veins; PVR: pulmonary vascular resistance; RPA: right pulmonary artery; RPVs: right pulmonary veins; SVC: superior vena cava

#### CMR

All examinations were performed using an Signa/GE CV/i 1,5 T scanner (maximal gradient = 40 mT/m, slew rate = 150 mT/m/sec) with a surface 4-channel cardiac phased-array coil (8-channel from 2005). A comprehensive CMR evaluation was performed following a previously published dedicated protocol [17]. Briefly, a preliminary 2D axial "Time of flight" (TOF) acquisition prepared with a stack of thin (3-mm) intersected slices covering the entire thorax from the aortic arch down to slightly below the diaphragm is prescribed. Further steps are: ECG-gated cine Steady State Free Precession (SSFP) sequences to visualize the ventricular/s anatomy and for quantitative assessment of ventricular dimensions, function, mass and stroke volume. SSFP sequences are also used to visualize the atrial septal defect, the ventricular septal defect and its relationship to the AV and VA valves, ventricular outflow tract/s and pulmonary branches. ECG-gated phase-velocity-contrast (PVC-CMR) free-breathing sequences perpendicular to the main pulmonary artery (if present), ascending aorta, pulmonary arteries, and systemic veins. Since 2005 the pulmonary vein flow was also assessed. PVC-CMR sequence parameters were: echo time: 3.7 ms; repetition time 6.2 ms; Flip Angle 20°; slice thickness 5 mm; field of view from 320 to 380 mm; matrix size 192 x140; trigger delay minimum; views per segment 2; Velocity encoding (VENC) according to excepted flow velocity. SSFP sequence parameters were: echo time: 1.4 ms; repetition time 3.5 ms; Flip Angle 60°, slice thickness 6-7 mm with no interslice gap; field of view from 320 to 380 mm; matrix size 224x224; trigger delay minimum; views per segment 8-14 according to heart rate, number of excitation 1 up to 3 if under sedation. The CMR exam was completed by a contrast-enhanced (gadopentate dimeglumine 0.4 ml/kg) three-dimensional Magnetic Resonance Angiography (MRA) finalized to the visualization (together with TOF acquisition) of the mediastinal vessels, as well as potential aorto-pulmonary and veno-venous collaterals; technical parameters were set as follows: echo time 1.1 ms; repetition time 3.5 ms; Flip Angle 20°, slice thickness 2.6 mm with no interslice gap; field of view from 320 to 380 mm and locs for slab 60; data matrix size 224 × 192; acquired to fill center lines and center slices of K-space first.

In patients studied in recent years the Late Gadolinium Enhancement (LGE) technique was added to the abovementioned protocol, in order to detect potential myocardial fibrous tissue [18]. In 36 patients < 8 years old and/or in patients who did not tolerate the CMR exam, it was performed with the patient deeply sedated by means of titrated propofol, and sequences were acquired as free-breathing.

Left and right pulmonary artery (LPA and RPA) diameter and area were calculated from MRA. The Nakata index [19], and Mac Goon index [20] were also respectively assessed as follows: LPA area + RPA area/BSA; LPA Ø + RPA Ø/Diaphragmatic Aorta Ø.

#### Cardiac catheterization

All procedures were performed using the Philips Integris H5000C Monoplane with the × Ray tube MRC 200 0508 ROT GS 1001. The anatomy of the pulmonary branches, aorta, superior and inferior vena cava and the aortic arch were evaluated by X-ray angiography in the AP plane and other appropriate planes if required, taking care to visualize potential aorto-pulmonary and/or veno-venous collaterals. A ventriculography was also performed to evaluate ventricular function. The pullback pressure was recorded in the pulmonary arteries, aorta, aortic arch, superior and inferior vena cava and in the ventricle. In case of suspected dynamic sub-aortic obstruction an Isoprenaline test was performed. Pulmonary vascular resistance and Mayo clinic index were calculated when possible. The latter was calculated as described [21]: adding pulmonary artery resistance to left ventricular end-diastolic pressure divided by Qp plus Qs where Qp and Qs are pulmonary and systemic blood flow respectively indexed to body surface area; The Nakata index [19], and Mac Goon index [20] were also respectively assessed as indicated above. Intra- and post-procedural complications were recorded. All procedures were performed with endotracheal intubation, under anesthesia.

The patients' history, physical examination, TTE and CMR data were analyzed by an experienced pediatric cardiologist (PF) and cardiac surgeon (BM) before performing CC and the patients were divided into two groups: Group 1: individuals with exhaustive pre-operative information in whom CC could be avoided ("CC not required" group); Group II: patients with indications for cardiac catheterization ("CC required" group).

Evaluation criteria for the patient group selection (hence for CC indication) were: 1) severe atrio-ventricular valve regurgitation semi-quantitatively evaluated by means of echo color Doppler 2) Ventricular EF < 50%. 3) Suspected pulmonary vein stenosis at echo and/or CMR. 4) Suspected systemic obstruction evaluated as follow: VSD flow velocity at echo-color Doppler > 2 m/s; aortic arch or isthmus narrowing < 40% of abdominal aorta diameter at CMR and/or flow velocity > 3 m/sec at echo-color Doppler. 5) Suspected pulmonary pathway or Glenn anastomosis obstruction evaluated as a diameter narrowing at MRA < 25% of the adjacent segment. 6) Complicated post-Glenn course: low cardiac output (defined as high or increasing plasma lactate and low mixed venous oxygen saturation) or prolonged pleural drainage (> 10 days). 7) Suspected significant veno-venous collaterals at MRA or when peripheral O2 saturation is less than 75%. 8) Presence of or suspected aorto-pulmonary collaterals at MRA and/or at echo. 9) Contradictory or incomplete clinical-instrumental findings. 10) Indications for interventional procedures.

By study design all patients of both groups underwent CC. (See Table 1 for demographic-clinical data and Table 2 for echo, CMR and CC data). Two patients evaluated in the last part of the study, categorized as group 1, were operated for Fontan without previous CC, according to the parents' wishes.

### Statistical analysis

Continuous data are expressed as mean ± SD and as median and range as appropriate. Inter-group comparisons were performed using the unpaired Student's t-test or Wilcoxon test, depending upon the normality of the data. A p-value < 0.05 was considered significant. The Bland-Altman method was used to determine the agreement between CC and CMR to assess Nakata and Mac Goon indexes. A correlation coefficient was used to determine the agreement between CC and CMR to assess Nakata and Mac Goon indexes.All data were analyzed by MedCalc v.7.3.0.1.

## Results

The CMR exam was completed in all patients but two: in one patient MR angiography could not be performed due to the impossibility of vascular access, in the other the CMR exam was interrupted due to cough and desaturation. No complications occurred.

**Figure 1:**
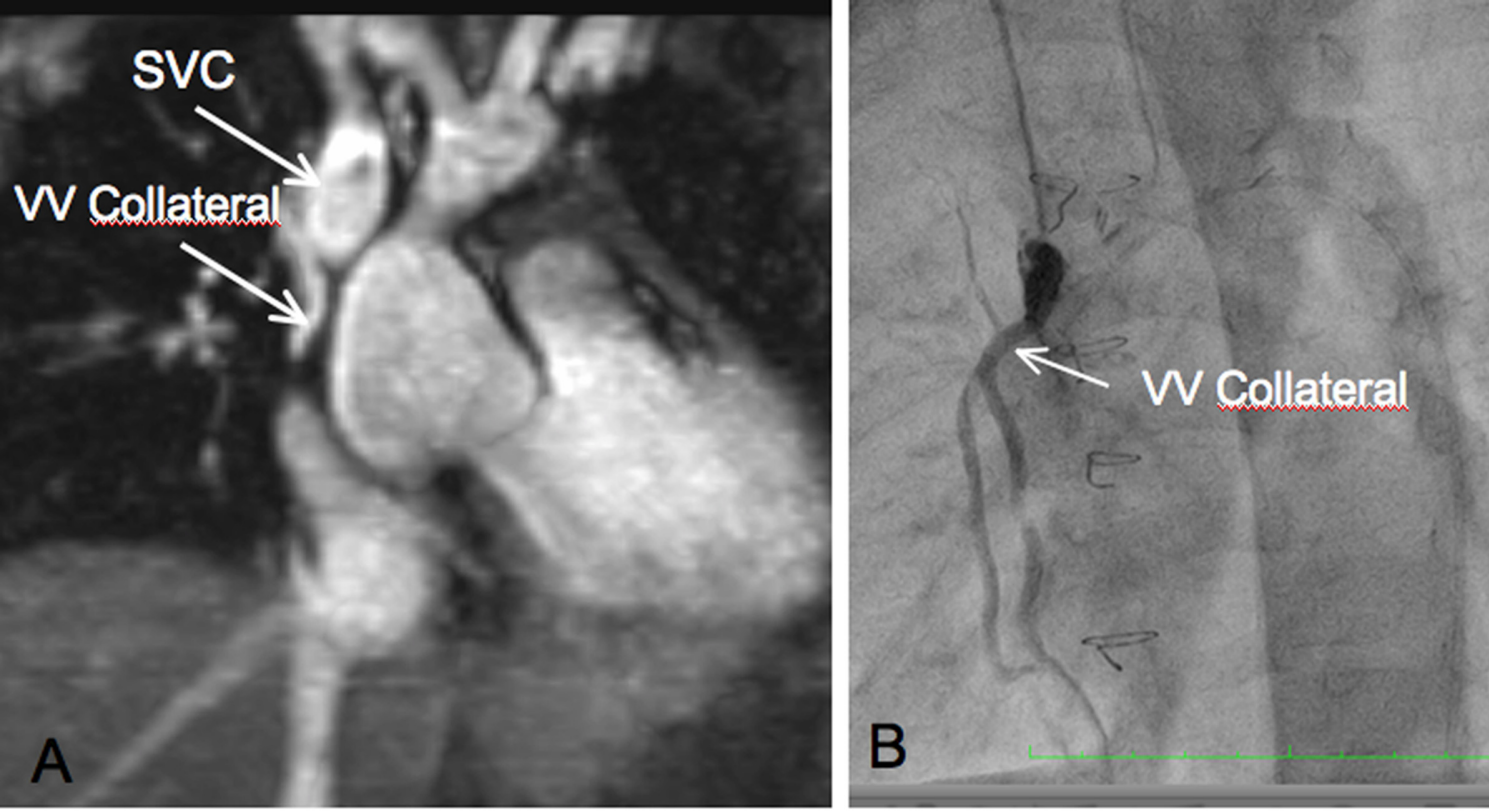
**Veno-venous collateral in a patient candidate for Fontan**. A: CMR: MIP reconstruction from TOF acquisition showing a veno-venous collateral from the SVC. B. Selective X-ray angiography in the veno-venous collateral.

According to history, echocardiographic and CMR data and before performing CC, 18 patients were considered eligible for Fontan without CC, and comprised Group I. In the other 26 patients CC was indicated (Group II) due to one of several of the following features: ventricular dysfunction (four patients), severe atrio-ventricular valve regurgitation (five), significant veno-venous collaterals (two), systemic obstruction (four), pulmonary artery hypoplasia (six) and suspected pulmonary vein stenosis (three). In one adult patient with complex congenital heart disease who had come from abroad with no medical records, CC was indicated because of lack of exhaustive clinical/instrumental information, and in another patient because of incomplete CMR data due to technical problems. Finally in six patients an interventional procedure was planned: one aortic coarctation balloon angioplasty; one pulmonary bifurcation balloon angioplasty; one LPA balloon angioplasty; one shunt embolization; two significant venous collateral to the atrium embolization (see Table 3 Figure 1). No Group I patient underwent an interventional procedure.

**Table 3:** Percutaneous interventional procedures

Diagnosis	Indication	Type of procedure
HLHS	Trans-isthmus gradient > 20 mmHg	Balloon angioplasty
DILV	SVC-LPA 2 mmHg gradient	LPA balloon angioplasty
TA	Significant LPA stenosis at X-ray angiography	LPA balloon angioplasty
PAIVS	Veno-venous collateral draining into the CS	Embolization
TA	Veno-venous collateral draining into the atrium	Embolization
Complex 2 ventricles	BTm shunt center opened at previous surgery	Embolization

Legend: mBT Modified Blalock-Taussig; CS: coronary sinus; DILV: double inlet left ventricle; HLHS: hypoplastic left heart syndrome; LPA: left pulmonary artery; PAIVS: pulmonary atresia intact ventricular septum; SVC: superior vena cava; TA: tricuspid atresia.

In all study population no major complication linked to the CC occurred. However a few minor complications were recorded: occlusion of the femoral artery in three patients, and a dissection of a small aorto-pulmonary collateral occurred during angiography in one patient.

Radiation dose-Area product was not significantly different in the two groups (Median 20 Gycm^2^, range: 5-40 in group I vs. median 26.5 Gycm^2^, range 9-270 p = 0.37). The corresponding estimated lifetime attributable risk of fatal and non-fatal cancer, based on Ionizing Radiation Committee VII (BEIR VII) released in 2006 [22] for all combinations of age was on average 1 in 350 patient. Mean RPA and LPA pressure were slightly higher in Group II than in Group I.

The echo exam resulted complete in only 15 pts (34%), mainly due to inadequate mediastinal vessel visualization. Based on echo and before MR, only 10 pts (22%) could have been categorized as group I ("CC not required" Group). The correlation between Nakata and Mac Goon indexes calculated by CC and CMR was respectively: r = 0,79, p < 0.001 and r = 0.76. P < 0.001. Agreement evaluated by Bland Altman analysis between Nakata and Mac Goon indexes calculated by CC and CMR was respectively : bias = 0.086, 95% limits of agreement = -74.6804 to 1.4540 and bias = -36.6, 95% limits of agreement = -0.06252 to 0.2361

TTE, CMR and hemodynamic data and differences between the two groups are summarized in Table 2.

Outcome: In Group I patients ("CC not required" Group) CC did not add any unexpected or useful information affecting surgical planning or indication. No interventional procedure was performed and all 18 patients underwent a total cavo-pulmonary anastomosis. The Fontan baffle was fenestrated in two patients due to long bypass time; additional surgical atrio-septectomy was performed in four patients.

**Figure 2:**
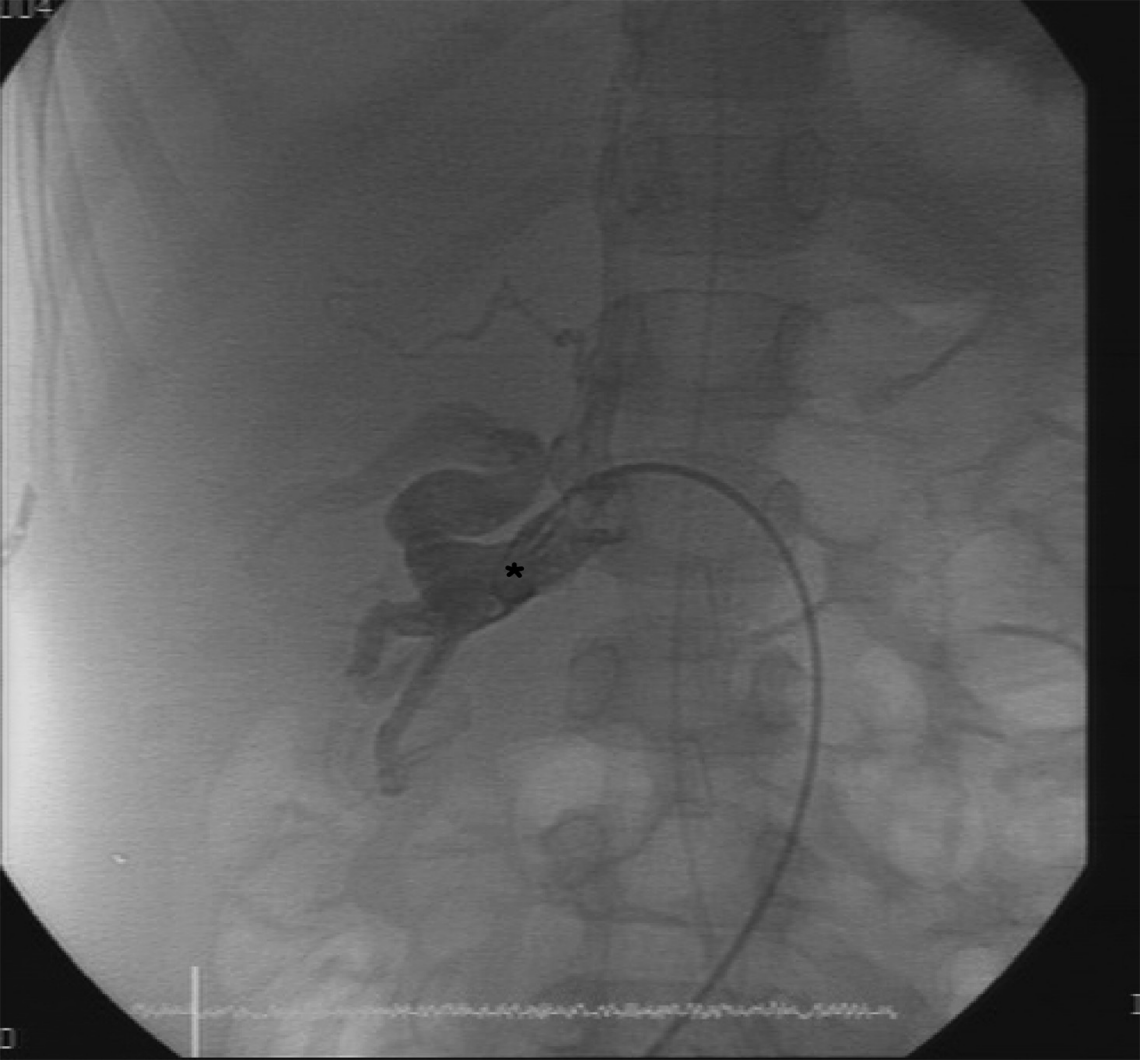
**Azygos-portal vein collateral in a patient candidate for Fontan**. X-ray angiography showing an Azygos-portal vein collateral (*) missed at MRA.

In Group II ("CC required" Group) new findings were observed at CC in three patients: a small coronary fistula, a significant venous collateral (Figure 2), and an aorto-pulmonary collateral, all missed by MRA. In one patient a stenotic pulmonary venous collector was diagnosed by MRA, not confirmed by CC, but confirmed and corrected by the surgeon. In the remaining patients, no unexpected findings were revealed by X-ray angiography; however, due to the presence of several risk factors that could interfere with Fontan surgical planning and prognosis, CC pressure data helped the decision-making process. As a matter of fact, four patients from this group were excluded from Fontan due to severe ventricular dysfunction, high pulmonary pressure, and severe pulmonary artery hypoplasia in the last two; 19 patients underwent total cavo-pulmonary anastomosis and in 13 of them the Fontan conduit was fenestrated. Additional surgical procedures were performed in 7 of them: two AV valve plasty, two enlargements of the pulmonary venous collector, one closure of right AV valve and pulmonary artery enlargement, one right AV valve closure and coronary fistula occlusion, and one Damus-Kaye-Stansel procedure. Two patients are awaiting Fontan at the time of writing, because they are still in very good clinical condition. One adult patient refused the intervention.

### Follow-up

37 patients underwent the Fontan operation (18 from group I, 19 from group II); 4 of them coming from abroad were lost at follow-up; the remaining 33 patients had a mean follow-up of 4.4 years (range 0.1-8.0). Mean peripheral oxygen saturation evaluated at the last ambulatory control was 95 ± 3.9%. In three of them peripheral oxygen saturation was < 90% and all are group II patients, as well as three patients who experienced an adverse outcome: 1 atrial major arrhythmia, 1 reduction of functional capacity due to low cardiac function, and 1 increased common AV regurgitation leading to surgical replacement. One patient from group I presented a neurological event due to thromboembolic complication. Finally two patients underwent successful percutaneous closure of the conduit fenestration. Surgical date, ICU stay, post-operative complications, follow-up and differences between the two groups are summarized in Table 4.

**Table 4:** Fontan and post-Fontan data

	All n 37/43	Group 1 n 18/18	Group 2 n 19/26	P
Fenestrated Fontan	15 (37%)	2 (11%)	13 (69%)	< 0.001
Additional procedure	11 (30%)	4 (22%)	7 (37%)	0.1
ECC time (min)	131 ± 59	108 ± 42	164 ± 65	0.003
ICU stay time (days)	3 (1-22)	2 (2-20)	4 (1-22)	0,06
*Peri-op Complications:*				
-Low cardiac output	6 (16%)	1 (5%)	5 (26%)	0.1
-Effusion	14 (38%)	7 (39%)	7 (37%)	0.8
- Neurologic events	3 (8%)	1 (5%)	2 (10%)	0.9
-Other complications*	4 (19%)	3 (17%)	1 (5%)	0.5
02 sat. at discharge	92 ± 3.9	95 ± 2.3	90 ± 3.6	<0.001
Follow-up (years)	4.4 ± 2.2	4.4 ± 2.6	4.3 ± 2	0.8

02% sat. at Follow-up	95 ± 4	97 ± 1.6	93 ± 4.7	0.001

* two diaphragmatic paralyses; one major bleed; one major atrial arrhythmia

Legend: ECC: extracorporeal circulation; ICU: intensive care unit

## Discussion

To our knowledge this is the first study where the diagnostic value of CC has prospectively been tested in a pre-Fontan setting. In our study 18 patients (41% of the entire population) have been considered eligible for Fontan without prior CC. In 16 of them the CC did not add any unexpected insight and the surgical planning did not change after it. In the other two the CC was not performed in accordance with the parents' wishes and they went to Fontan directly without prior CC. In all 18 patients the surgical procedure resulted uneventful as did the short- term follow-up. At medium-term a neurological sequel, not due to an incorrect diagnosis but rather to coagulation mismanagement, was the only complication occurring in one patient from group I. Of note is also that at medium-term follow-up (4.4 ± 2.6 years) mean oxygen peripheral saturation was 97% ± 1.6 and no additional procedure was prescribed.

Conversely, CC was indicated in 26 patients (59% of the whole population study) prior to Fontan, according to the abovementioned criteria. This percentage is in concordance with similar studies that retrospectively evaluated the diagnostic value of CC (10-11). New features were found in three patients. In the remaining patients, although no unexpected findings emerged from X-ray angiography, CC pressure data helped the decision-making process. In particular patients with several risk factors that interfere with Fontan surgical planning and prognosis, according to the algorithm proposed in the present study, enter in the "CC required" group such as the patients excluded from Fontan in our study. In recent years contraindication for Fontan is a relatively rare condition (10% in our population) and is generally based on several parameters. At our institution exclusion from Fontan is generally multifactorial and in these cases, we believe that CC could add more information and thus aid a difficult decision-making process. Our results indicate that in association with more traditional clinical, ECG and echocardiographic data, the routine use of CMR before the Fontan procedure is of pivotal importance. With this integrated imaging approach, CC could be avoided in selected patients.

The aim of the diagnostic evaluation before Fontan is to identify those few patients in whom the Fontan operation should not be performed, and those who require additional intervention before or at the time of Fontan. From the literature, whether CC is necessary before the Fontan operation remains controversial, especially when sufficient data are provided by echocardiography and CMR. The main arguments in favor of routine CC before Fontan are assessment of pulmonary artery pressure and vascular resistance [4] as important prognostic factors [20,23-25]. Either potential collateral veins between the superior vena cava and the left atrium or peripheral pulmonary artery stenosis are also a potential CMR caveat [4]. Moreover, CMR fails to visualize very small aorto-pulmonary collaterals that, according to some groups, are correlated with worse outcome after Fontan, and therefore are routinely embolized [26,27]. However these findings have not been reproduced by other studies [28] and at our institution, coil embolization of very small systemic to pulmonary collaterals is not routinely performed before Fontan. On the other hand, CMR's ability to assess pulmonary artery and vein flow could be a unique tool, suggesting the amount of artero-pulmonary collateral flow. Therefore we believe that CMR is emerging as a versatile new diagnostic imaging tool, able to give us accurate anatomical and functional details that are not otherwise available. With a non-invasive and radiation-free approach, CMR can assess ventricular function and potential scar tissue and pulmonary artero-venous flow more accurately than any other diagnostic tool.

Another issue in favor of avoiding CC in all Fontan candidates is that management strategy for patients with a functional single ventricle has evolved to include staging bidirectional cavopulmonary anastomosis in most cases and it has become uncommon to exclude patients from Fontan, based on catheterization data [29]. In these complex patients, if a standardized treatment has been correctly followed since birth, pulmonary resistance is seldom an important issue at the pre-Fontan stage. It is of note that all but one of the patients included in our study had already undergone assessment of pulmonary resistance by means of a pre-Glenn CC a few years before. Since pulmonary resistance does not usually change after the Glenn procedure, there is no reason to indicate CC before Fontan in such patients if only for assessing pulmonary resistance. From a practical point of view, pulmonary pressure issues can also be dealt with in the operating room in a pre-extracorporeal circulation setting in close collaboration with the cardio-anesthesiologist, cardiologist and cardiac surgeon. In cases of cavo-pulmonary anastomosis with additional antegrade pulmonary flow, pulmonary pressure can be assessed even better in the operating room than by traditional CC, due to the fact that with an open chest it is possible to modulate or even to stop the antegrade pulmonary artery flow.

The echo exam was considered complete in only 34% of the patients. This relatively low percentage, confirmed by the literature (11), is mainly due to the typically poor visualization of the mediastinal vessels.

### Clinical implications

**Figure 3:**
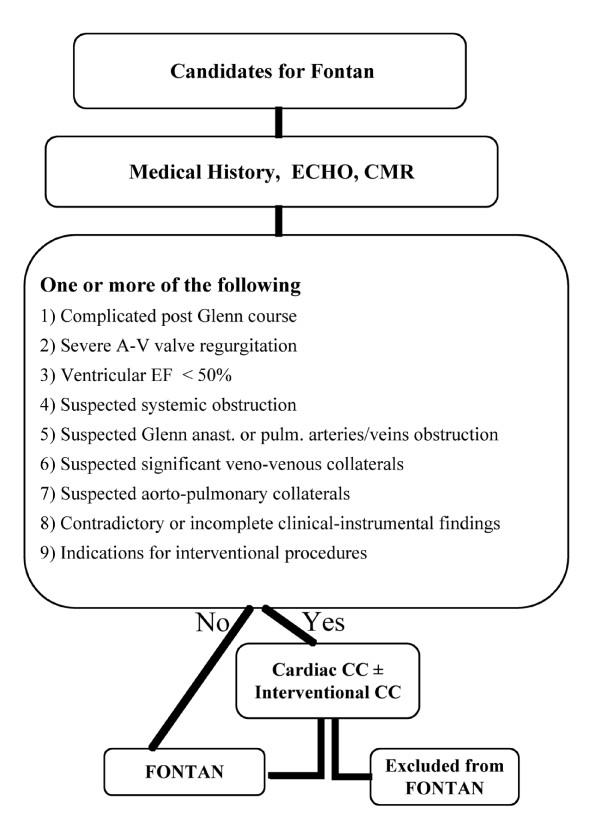
**Proposal diagnostic algorithm in patient candidates for Fontan operation**. Criteria are detailed in the text. AV: Atrio-Ventricular; MIP: Maximum Intensity Projection PVR: Pulmonary Vascular Resistance; SVC: Superior Vena Cava; TOF: Time of Flight; UVH: Univentricular Heart; VV: Veno-Venous.

Our results showed that CC is indicated only in selected cases before the Fontan operation. In clinical practice we propose a personalized diagnostic flow chart for evaluation of patients with functional UV heart, candidates for Fontan circulation (Figure 3). This is supported by better knowledge of the "non-physiologic" Fontan circulation, deriving from more than 35 years' experience [2] and the development of new diagnostic imaging tools, mainly CMR. We advocate CC in selected cases with historical or clinical/laboratory suspicion of high pulmonary vasculature pressure and/or resistance, including poor ventricular function and/or severe atrio-ventricular valve insufficiency; in case of evidence or suspected pulmonary pathway stenosis, or in case of systemic obstruction; and of course when any interventional procedure is indicated, as in case of suspected aorto-pulmonary or veno-venous major collaterals, especially if potentially draining to the left atrium. The proposed algorithm could serve in selected cases to avoid a CC that, as an invasive diagnostic tool, presents some immediate procedural risks, as well as long-term risks linked to the radiation burden. However, the above-proposed flow chart should be personalized according to the institutional and CMR lab experience.

The routine use of pre-Fontan CC is based on over 50 years of clinical experience in CC data and x-ray. In fact, surgeons and cardiologists are more used to exploring the anatomy of the heart and vessels as well as the hemodynamics by means of x-ray angiography, rather than CMR, although the latter is far more informative. This will change in the near future as CMR becomes increasingly widespread and accepted, and as pediatric cardiology courses increasingly include MR tuition.

### Study limitation

This study is not a randomized study, and presents all the limits of a prospective, observational, single-center study, with a relatively low sample size. Nevertheless, the highly selected population, the homogeneous criteria of clinical decision-making, and access to state-of-the art technology and expertise may provide a valuable perspective for evaluating these challenging patients.

This study had not been designed to test echo performance in pre-Fontan patients, so the relatively low percentages of "complete echo" indicated above would have been slightly superior if the echo exam had been repeated by an experienced cardiologist, at least in a second attempt if considered "not complete".

## Conclusion

In a pre-Fontan setting, an accurate clinical history and instrumental evaluation based mainly on echo and targeted cardiac CMR can screen patients suitable for Fontan who could skip CC, avoiding the acute risks linked to invasive procedures and contrast-induced nephropathy. Perhaps equally important, a young patient will avoid the long-term cancer risks connected to radiation exposure and preserve vascular access to the heart. Following this flow chart in patients before Fontan, diagnostic CC becomes an optional procedure indicated in selected cases with known risk factors that could interfere with Fontan surgical planning and prognosis and when interventional CC is indicated. Moreover, since CMR can provide accurate anatomical and functional details unavailable via any other tool, the widespread use of this technique has great potential to offer more detailed knowledge of Fontan "physiology". However, since ours was a small, single-center study, and due to lack of long-term outcomes, further studies are needed to confirm the proposed algorithm.

## Competing interests

The authors declare that they have no competing interests.

## Authors' contributions

AL carried out the data collection, participated in the study design and performed the statistical analysis. DDM participated in CMR protocol set-up and optimization of CMR examinations. LS was the anesthesiologist focusing on CMR and catheterization studies. EP was involved in drafting the manuscript, ML helped draft the manuscript, BM participated in study design and reviewed all the CMR examinations. PF conceived the study, and participated in its design and coordination and helped draft the manuscript.

All authors read and approved the final manuscript.
